# Real-World Evolution of Prescribing Patterns and Safety Profiles of Biologic and Targeted Synthetic Therapies in Inflammatory Rheumatic Diseases: A Comparative Study Between 2018–2019 and 2023–2024

**DOI:** 10.3390/pharmaceutics18080983

**Published:** 2026-08-10

**Authors:** Antonio Fabiano, Lorenza Guarnieri, Domenico Frajia, Carmela Spinoso, Massimo L’Andolina, Angelica Profiti, Damiana Scuteri, Corrado L’Andolina, Francesca Bosco, Eugenio Donato Di Paola, Rita Citraro, Giovambattista De Sarro

**Affiliations:** 1 Department of Health Sciences, University “Magna Graecia” of Catanzaro, 88100 Catanzaro, Italy; 2 Rheumatology Outpatient Clinic, ASP Vibo Valentia-Tropea Hospital, 89861 Tropea, Italy; 3 Research Center FAS@UMG, Department of Health Science, University “Magna Graecia” of Catanzaro, 88100 Catanzaro, Italy

**Keywords:** rheumatoid arthritis, psoriatic arthritis, inflammatory rheumatic diseases, biologics, biosimilars, adverse events

## Abstract

**Background/Objectives**: Biologic and targeted synthetic disease-modifying antirheumatic drugs (b/tsDMARDs) have expanded treatment options for inflammatory rheumatic diseases, highlighting the need for continuous pharmacovigilance. This study evaluated changes in prescribing patterns and safety profiles of b/tsDMARDs between 2018–2019 and 2023–2024 in routine clinical practice. **Methods**: A retrospective observational study was conducted in patients with rheumatoid arthritis (AR), psoriatic arthritis (PsA), ankylosing spondylitis (AS), or juvenile idiopathic arthritis (JIA) treated at a rheumatology outpatient clinic in Southern Italy. Demographic and clinical characteristics, prescribed therapies, treatment switches/swaps, therapeutic failures, and adverse events (AEs) were collected through a structured pharmacovigilance program. Prescribing trends between the two study periods were compared using chi-square analysis. **Results**: A total of 342 patients were included (202 in 2018–2019 and 140 in 2023–2024). Prescribing patterns changed significantly over time (χ^2^ = 83.24, *p* < 0.0001), with increased use of newer therapeutic classes and biosimilars, while tumor necrosis factor (TNF) inhibitors remained the most frequently prescribed drugs. The proportion of biologic-naïve patients increased from 52.0% to 66.4%, whereas AEs decreased from 31.7% to 15.0%, with no serious adverse events (SAEs) reported. **Conclusions**: Prescribing strategies for inflammatory rheumatic diseases evolved substantially between 2018 and 2024, reflecting the availability of new therapeutic options and a more personalized treatment approach. b/tsDMARDs showed a favorable real-world safety profile, supporting the importance of ongoing pharmacovigilance.

## 1. Introduction

Rheumatic diseases are common and often chronic conditions characterized by immune-mediated alterations that lead to inflammation and damage affecting the musculoskeletal system and, in some cases, internal organs. Early diagnosis and targeted therapeutic intervention are crucial to limit their long-term consequences [1].

The introduction of biologic and targeted synthetic disease-modifying antirheumatic drugs (b/tsDMARDs) has revolutionized the management of rheumatic diseases, leading to substantial improvements in both prognosis and quality of life [2]. However, the immunomodulatory effects of these agents necessitate structured safety monitoring in real-world clinical settings, beyond the constraints of pivotal registration trials, thereby highlighting the pivotal role of observational pharmacovigilance. Disease-modifying antirheumatic drugs (DMARDs) are currently classified into conventional synthetic, biologic (including biosimilar), and targeted synthetic agents [3]. Through modulation of the immune system, all DMARD classes are associated with an increased risk of serious infections, tuberculosis reactivation, opportunistic infections, and potentially malignancies. Beyond these shared safety concerns, individual therapeutic classes may present specific adverse event (AEs) profiles, including injection or infusion reactions with biologic agents, gastrointestinal and mucocutaneous manifestations with interleukin (IL) inhibitors, and hematological, cardiovascular, or thromboembolic risks reported with Janus (JAK) kinase inhibitors [4,5,6].

National and international registries have therefore become essential tools for assessing the long-term effectiveness and safety of these therapies in large real-world cohorts comprising thousands of patients. Prospective cohort studies and national or regional registries enable the estimation of AEs incidence rates, the identification of risk factors (such as age, comorbidities, and concomitant corticosteroid use), and the comparison of safety profiles across different therapeutic classes and individual agents Frisell [6,7,8].

Active regional and national pharmacovigilance programs have demonstrated the ability to detect rare AEs, identify emerging safety signals, and highlight differences among individual agents and biosimilars [5,7,8,9].

Overall, observational studies and pharmacovigilance registries indicate that biologic and targeted therapies exhibit generally acceptable safety profiles in rheumatic diseases, although specific risks vary according to the therapeutic class and individual agent. Therefore, structured post-marketing surveillance is essential to characterize long-term safety, identify unexpected safety signals, and support personalized therapeutic decision-making in real-world clinical practices [10,11,12].

The use of b/tsDMARDs has increased substantially over time, while the interval between diagnosis and treatment initiation has progressively shortened [13,14].

Currently available b/tsDMARDs include TNF inhibitors, Interleukin-6 (IL-6) inhibitors, β-cell–targeted therapies, T-cell co-stimulation modulators, and JAK inhibitors, which act through different mechanisms of immune modulation. Their use is recommended according to international guidelines in patients with inadequate response to conventional synthetic DMARDs, with treatment choice based on disease characteristics and patient-related risk factors [15]. Although these therapies are associated with high costs, their accessibility depends on national healthcare systems and reimbursement policies. In Italy, access to these treatments is regulated by the Italian Medicines Agency (AIFA) reimbursement criteria [16].

In addition, a shift in prescribing patterns has been observed, characterized by a reduced use of TNF inhibitors as first-line biologic therapy and an increased adoption of other therapeutic classes, including IL-6 inhibitors, Janus kinase (JAK) inhibitors, and biosimilars [17].

Data derived from previous regional pharmacovigilance programs, including our own previously published studies, indicate that most patients do not experience serious adverse events (SAEs), and that the majority of treatment switches are driven by lack of efficacy rather than drug-related toxicity [10,17].

The aim of this study was to describe changes in prescribing patterns of b/tsDMARDs between 2018–2019 and 2023–2024 and to evaluate the occurrence of reported AEs, treatment ineffectiveness, and treatment switching in a real-world rheumatology cohort.

By providing real-world evidence on prescribing trends and the safety profile of b/tsDMARDs, this study aims to support more informed therapeutic decision-making, helping clinicians tailor treatment according to the risk of AEs in specific patient populations and ultimately improving patient care.

## 2. Materials and Methods

### 2.1. Study Design and Data Collection

This study analyzed data from patients who met the inclusion criteria and received b/tsDMARD therapy during the periods 2018–2019 and 2023–2024 at the Rheumatology Unit of General Medicine of Tropea Hospital (Provincial Health Authority, ASP, Vibo Valentia, Calabria, Italy). Demographic and clinical characteristics, treatment prescriptions, treatment discontinuations, therapeutic failures, treatment switches, and AEs were collected for each patient. Data were collected and managed using a dedicated Microsoft Excel database developed by clinical pharmacology specialists in accordance with Good Clinical Practice guidelines. These activities were coordinated by the Regional Drug Information Center of Calabria, which was established approximately 20 years ago as part of a research project funded by the Italian Ministry of Health under the Special Programmes (Art. 12-bis, paragraph 6, Legislative Decree No. 229/1999), entitled “Farmaci e fitoterapici, impatto sulla salute, vigilanza e sviluppo di un modello organizzativo interregionale” (“Medicinal Products and Herbal Medicines: Health Impact, Surveillance, and Development of an Interregional Organizational Model”) [18]. The study involved physicians with a specialization in Clinical Pharmacology, who were responsible for pharmacovigilance activities, database management, AEs assessment, and follow-up interviews. To ensure patient confidentiality, each participant was assigned a unique encrypted identification code, in compliance with the Declaration of Helsinki (1964) and its subsequent amendments. The study protocol was approved by the Calabria Regional Ethics Committee (protocol no. 278/2015 and protocol no. 387, approved on 19 November 2020). For each enrolled patient, the following information was recorded: age, sex, diagnosis, disease duration, current or previous corticosteroid therapy, biologic or biosimilar treatments, conventional DMARDs, concomitant medications, relevant comorbidities, treatment discontinuations or switches and their underlying reasons, primary or secondary treatment failure, and the occurrence of AEs. The occurrence of AEs was defined as any unfavorable medical event experienced during treatment with a biologic agent, irrespective of its causal relationship with the therapy. Events were classified as SAEs if they resulted in death, were life-threatening, required hospitalization or prolongation of hospitalization, caused persistent or significant disability, or were associated with congenital anomalies/birth defects [19].

### 2.2. Inclusion Criteria and AEs Assessment

All patients enrolled in this study fulfilled the following inclusion criteria: age ≥ 18 years; past diagnosis of Rheumatoid arthritis (RA), Psoriatic arthritis (PsA), Juvenile idiopathic arthritis (JIA) or Anchylosing spondylitis (AS); starting treatment with one b/tsDMARD and having a treatment and follow-up duration of at least 12 months. Informed consent was obtained from all enrolled patients at the time of admission. The index date was defined as the initiation of b/tsDMARD therapy according to the study protocol for each patient, including both b/tsDMARD-naïve individuals and those previously treated with a b/tsDMARD. Regarding clinical follow-up, patients were routinely evaluated at the specialist clinic every 3 months; however, they were promptly examined before the scheduled appointment if any suspected adverse event occurred. A hybrid system was adopted to detect side effects during the follow-up period starting from the index date, combining telephone interviews conducted by the pharmacologist, as previously described, with routine specialist visits [20].

In some cases, patients who initially benefit from treatment may subsequently lose responsiveness or develop adverse effects over time. In our program, the reasons for treatment discontinuation (with or without switching therapy) included primary or secondary therapeutic failure—defined as lack of response within 16 weeks or absence of efficacy of the newly prescribed b/tsDMARD—or the occurrence of AEs. For each AE identified, the attending physician completed the AIFA reporting form for suspected adverse drug reactions. All AEs were categorized according to the Medical Dictionary for Regulatory Activities (MedDRA) classification system, documenting details such as onset time, resolution, severity, and outcome. All reported AEs were evaluated and validated by the Regional Drug Information Center before being submitted to the Italian National Pharmacovigilance Network (RNF).

When AEs were considered serious, particularly in potentially life-threatening situations, discontinuation of the current b/tsDMARD treatment and transition to another b/tsDMARD became necessary. The term switch refers to the substitution of one b/tsDMARD with another agent belonging to the same therapeutic class, whereas changing to a b/tsDMARD from a different class is defined as a swap. Currently, the concept of switching also includes replacing an originator biologic (reference product) with its corresponding biosimilar. Biosimilars are highly comparable versions of complex biologic drugs that provide similar efficacy and safety profiles while generally being available at lower costs.

### 2.3. Statistical Analysis

The sample size was determined based on consecutive patients admitted to the study center during the study period and was not predefined (i.e., non-randomized enrollment). A descriptive analysis was performed to summarize the baseline and demographic characteristics of patients at the index date. Continuous variables included age, disease duration, and time from treatment initiation to treatment discontinuation or occurrence. Categorical variables included sex, diagnosis, previous or current corticosteroid therapy, biologic or biosimilar treatment, concomitant conventional DMARDs, comorbidities, treatment discontinuation, treatment switches, therapeutic failure, and AEs. Variables were classified according to their measurement scale and analyzed accordingly [21]. Baseline and demographic characteristics of patients at the index date were summarized using descriptive statistics. Continuous variables were reported as mean ± standard deviation (SD) or median (interquartile range, IQR; 25th–75th percentile), whereas categorical variables were expressed as absolute frequencies and percentages.

Each patient received the biologic treatment selected by the treating rheumatologist according to routine clinical practice and current rheumatology guidelines. Treatment selection was based on the physician’s clinical judgment, considering disease characteristics, previous treatment history, comorbidities, and therapeutic response. No treatment assignment was performed by the study investigators, as this was an observational study.

The distribution of prescriptions among different biologic drug classes was compared between the study periods using a chi-square (χ^2^) test of independence on contingency tables. The chi-square (χ^2^) test of independence is a statistical test used to determine whether there is a significant association between two categorical variables by comparing the observed frequencies with the frequencies expected under the assumption of independence. In this study, the test was used to evaluate whether the distribution of prescribed biologic drug classes differed between the two study periods. This analysis was relevant to identify possible changes in treatment patterns over time [22].

The analysis was performed to assess the association between the study period and the distribution of prescribed b/tsDMARD therapies. In this regard, it is worth noting that the study populations represent dynamic real-world cohorts; a subset of patients (31%) enrolled in the 2023–2024 period was also present in the 2018–2019 cohort due to ongoing follow-up or therapeutic switching. The rest of the cohort shifted due to real-world dynamics, including deaths, treatment discontinuations, and patient migration to other clinical centers or regions (e.g., due to regional variations in the prescriptive availability of newer b/tsDMARDs). Therefore, to strictly satisfy the requirements of the chi-square (χ^2^) test for comparing prescribing patterns, the statistical unit of analysis was defined as the individual treatment initiation event rather than the patient, treating these events as independent cross-sectional snapshots of clinical practice in each specific historical period. A *p*-value < 0.05 was considered statistically significant.

## 3. Results

### 3.1. Demographic and Clinical Characteristics of the Study Cohorts

Baseline demographic characteristics and prescribing patterns were analyzed to contextualize the evaluation of treatment changes, possible AEs, and treatment ineffectiveness across the two study periods. Table 1 and Table 2 report the general characteristics of patients included in this study across the two corresponding time periods (2018–2019 and 2023–2024).

During the first study period (2018–2019), 202 patients (125 females and 77 males; mean age at first biologic therapy 43.7 ± 15.6 years) initiated biologic treatment for RA (*n* = 91, 45.05%), PsA (*n* = 104, 51.48%), and spondyloarthritis (SpA; *n* = 7, 3.46%) at the Rheumatology Outpatient Clinic of Tropea Hospital, ASP Vibo Valentia, Italy. During this period, no patients with JIA were treated in the clinic (Table 1).

During the second study period (2023–2024), 140 patients (90 females and 50 males; mean age at first biologic therapy 48.1 ± 9.5 years) initiated advanced therapy (bDMARDs or tsDMARDs) at the same outpatient clinic for RA (*n* = 64, 45.71%), PsA (*n* = 73, 52.14%), JIA (*n* = 1, 0.71%), and SpA (*n* = 2, 1.43%) (Table 2).

In both study cohorts, the most prevalent comorbidity was hypertension (48.5% in 2018–2019 and 50.71% in 2023–2024), followed by cardiovascular diseases in the 2018–2019 cohort and fibromyalgia in the 2023–2024 cohort. In the first study period, 52% of patients were biologic-naïve, whereas in the second period this proportion increased to 66.43% (Table 1 and Table 2).

During the 2018–2019 period, the most frequently prescribed biologic agent at index date was etanercept (ETN) (*n* = 38; 18.81%), followed by adalimumab (ADA) (*n* = 34; 16.83%), abatacept (ABT) (*n* = 27; 13.36%), tocilizumab (TCL) (*n* = 24; 11.9%), infliximab (IFX) (*n* = 23; 11.4%), golimumab (GOL) (*n* = 21; 10.4%), certolizumab (CERT) (*n* = 11; 5.4%), secukinumab (SEC) (*n* = 11; 5.4%), and ustekinumab (UST) (*n* = 6; 3%).

In the 2023–2024 period, ETN remained the most commonly prescribed agent (*n* = 30; 21.4%), followed by ABT (*n* = 13; 9.3%), tofacitinib (TFC) (*n* = 13; 9.3%), GOL (*n* = 12; 8.6%), ADA) (*n* = 10; 7.1%), SEC (*n* = 10; 7.1%), baricitinib (BRC) (*n* = 6; 4.3%), CERT (*n* = 6; 4.3%), ixekizumab (IXK) (*n* = 6; 4.3%), upadacitinib (UPC) (*n* = 6; 4.3%), guselkumab (GUS) (*n* = 5; 3.6%), TCL) (*n* = 4; 2.9%), risankizumab (RSK) (*n* = 2; 1.4%), rituximab (RTX) (*n* = 2; 1.4%), sarilumab (SAR) (*n* = 1; 0.7%), and IFX (*n* = 1; 0.7%).

Overall, the use of biosimilars increased over time. Infliximab biosimilars were the most frequently prescribed biosimilars in the 2018–2019 period (*n* = 4; 2%), whereas adalimumab and etanercept biosimilars were the most used in the 2023–2024 period (*n* = 6; 4.3%).

The drug-specific data are reported in Table 3 and Table 4. Table 3 and Table 4 also list the most frequently prescribed concomitant treatments, and in agreement with our previously published findings [20], methotrexate (MTX) was confirmed as the most commonly prescribed concomitant medication in this study as well.

**Figure 1:**
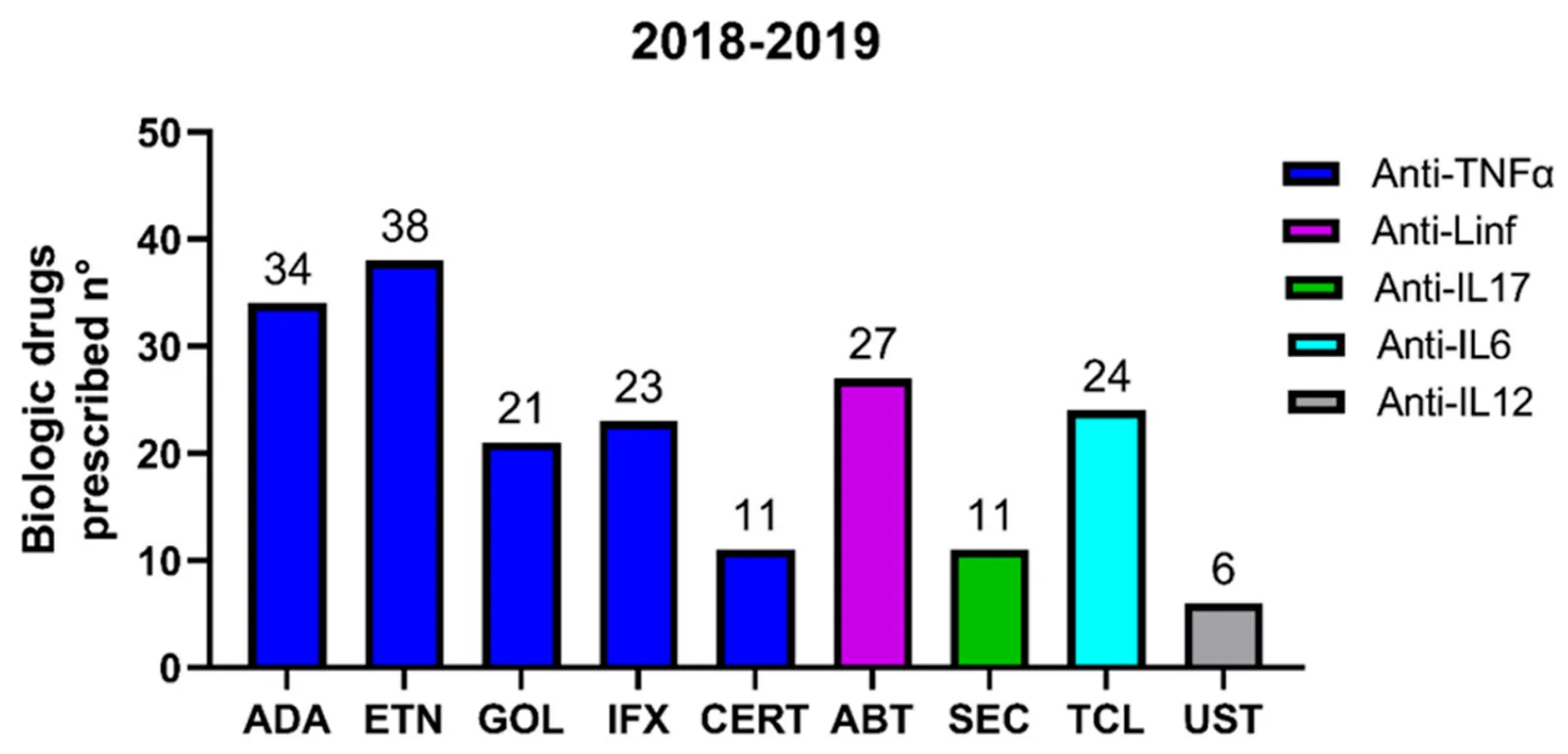
Prescribing patterns of biologic therapies in patients treated for rheumatic diseases at the Rheumatology Unit of Tropea Hospital (Calabria, Italy) during the 2018–2019 period. Abbreviations: ADA, Adalimumab; ETN, Etanercept; GOL, Golimumab; IFX, Infliximab; CERT, Certolizumab; ABT, Abatacept; SEC, Secukinumab; TCL, Tocilizumab; UST, Ustekinumab.

**Figure 2:**
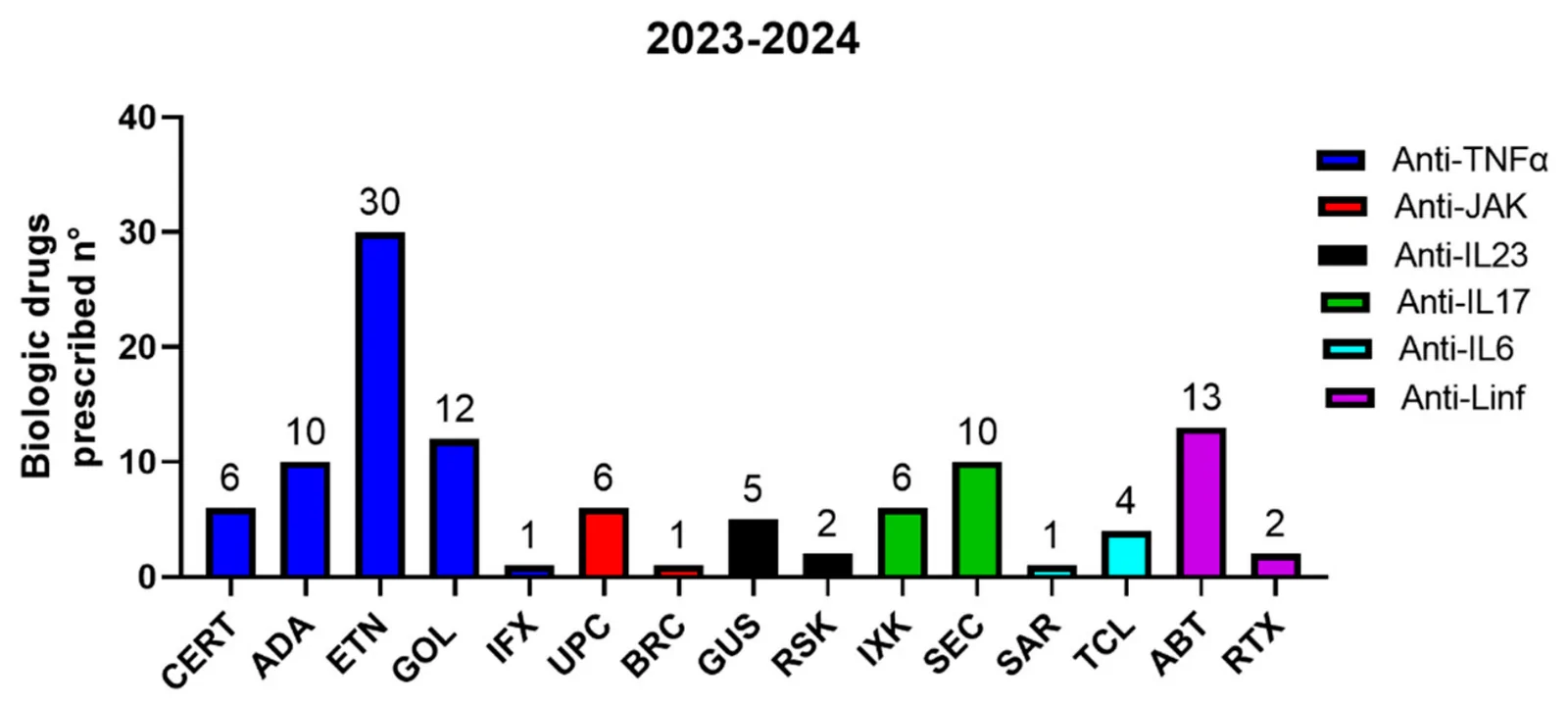
Prescribing patterns of b/tsDMARD therapies in patients treated for rheumatic diseases at the Rheumatology Unit of Tropea Hospital (Calabria, Italy) during the 2023 to 2024. Abbreviations: CERT, Certolizumab; ADA, Adalimumab; ETN, Etanercept; GOL, Golimumab; IFX, Infliximab; UPC, Upadacitinib; BRC, Baricitinib; GUS, Guselkumab; RSK, Risankizumab; IXK, Ixekizumab; SEC, Secukinumab; SAR, Sarilumab; TCL, Tocilizumab; ABT, Abatacept; RTX, Rituximab.

Figure 1 and Figure 2 illustrate the prescribing patterns of biologic therapies in patients treated for rheumatic diseases at the Rheumatology Unit of Tropea Hospital (Calabria, Italy) during the 2018–2019 and 2023–2024 periods, respectively.

To facilitate interpretation of the figures, drugs targeting the same molecular pathway are displayed using the same color code.

**Figure 3:**
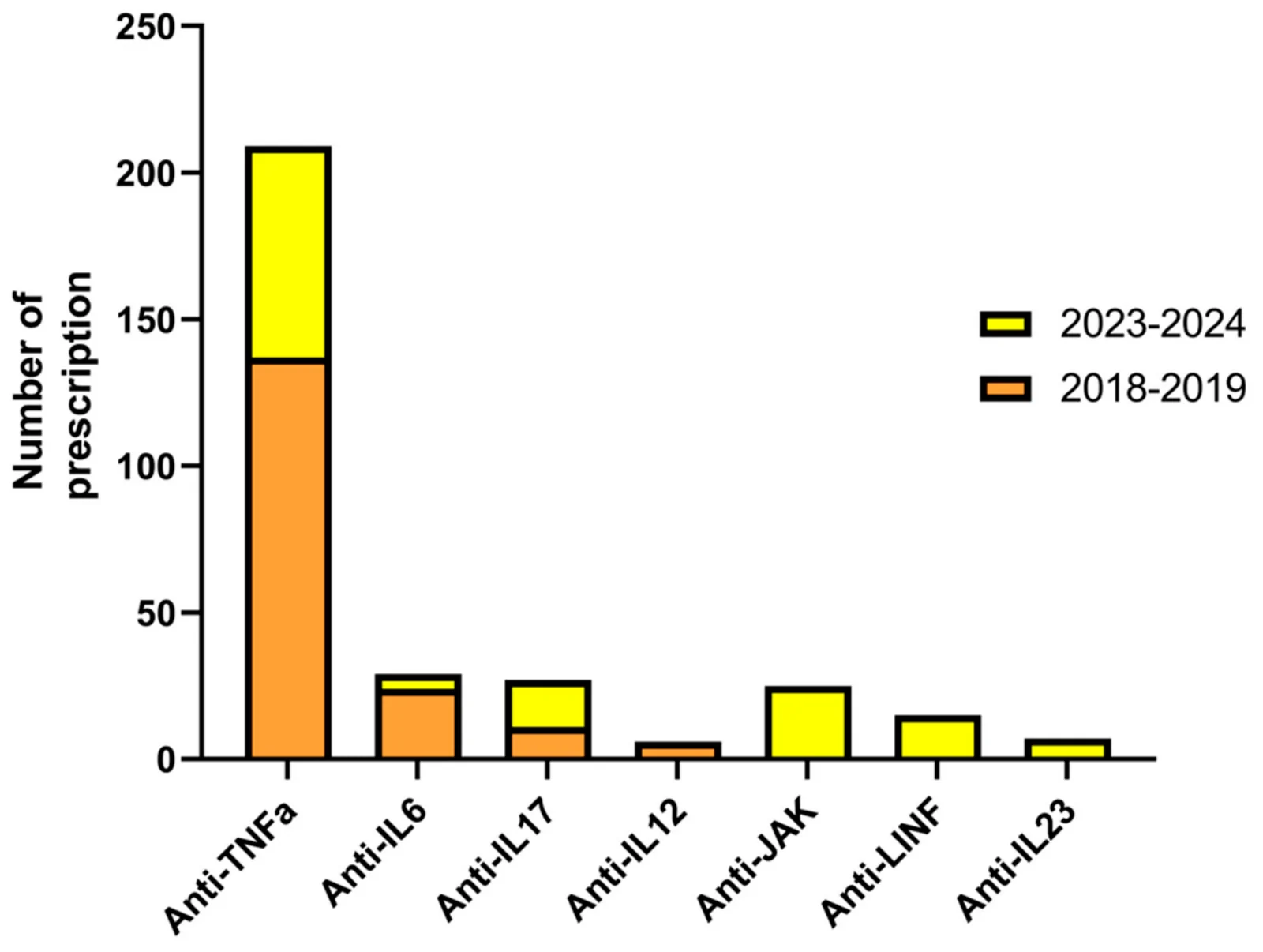
Comparation of b/tsDMARD prescription by therapeutic class between 2018–2019 and 2023–2024 periods. Bars represent the number of prescriptions recorded for each drug class. A significant difference in prescription distribution was observed between the two periods (χ^2^ = 83.24, df = 6, *p* < 0.0001).

Figure 3 highlights the change in prescribing trends between the two study periods included in the analysis. The distribution of biologic prescriptions differed significantly between 2018 and 2023 (χ^2^ = 83.24, df = 6, *p* < 0.0001). While TNF-α inhibitors remained the most frequently prescribed b/tsDMARDs, their relative contribution decreased over time. Conversely, an increased use of Interleukin-17 (IL-17) inhibitors and Janus kinase inhibitors was observed, together with the emergence of Interleukin-23 (IL-23)-targeted therapies. These findings indicate a substantial shift in prescribing patterns and a progressive diversification of available therapeutic options.

### 3.2. Switches and Swaps Frequencies Among bDMARDs

Treatment switches and swaps were evaluated as indicators of possible treatment ineffectiveness or tolerability issues. Table 5, Table 6, Table 7 and Table 8 provide a detailed overview of all treatment switches and swaps. For the study periods considered (Table 5 and Table 6, 2018–2019; Table 7 and Table 8, 2023–2024), these tables report the number of patients who underwent a switch or swap from the initially selected biologic therapy.

### 3.3. Characteristics of AEs

The occurrence of reported AEs and treatment ineffectiveness represented the primary pharmacovigilance outcomes evaluated in the present study. Treatment switches/swaps were analyzed according to their underlying causes, distinguishing between ineffectiveness and AEs. During the 2018–2019 study period, 64 AEs (31.68%), 0 SAEs, and 27 cases of ineffectiveness (13.4%) were recorded. In the 2023–2024 period, 21 AEs (15%), 0 SAEs, and 27 cases of ineffectiveness (19.3%) were observed. Overall, a lower frequency of reported AEs was observed in the 2023–2024 cohort compared with the 2018–2019 cohort; potential reasons for this finding are discussed in the Section 4.

The 64 AEs reported in the 2018–2019 cohort were associated with ADA (16/64; 25%), ETN (14/64; 21.9%), GOL (6/64; 9.4%), IFX (16/64; 25%), ABT (2/64; 3.1%), CERT (4/64; 6.2%), and TCL (6/64; 9.4%), whereas no AEs were observed with SEC or UST.

The 21 AEs observed in the 2023–2024 cohort were related to ABT (3/21; 14.3%), ADA (5/21; 23.8%), CERT (2/21; 9.5%), ETN (4/21; 19%), IXK (2/21; 9.5%), and TFC (4/21; 19%), while no AEs were reported with BRC, GOL, GUS, IFX, RSK, RTX, SAR, SEC, or TCL during this period.

The most frequently reported AEs included asthenia, injection-site reactions, and skin disorders, consistent with findings from our previously published larger study [20]. In the 2018–2019 cohort, AEs were most frequently associated with IFX (69.6% of total prescriptions) and ADA (47% of total prescriptions) (Table 9), whereas in the 2023–2024 cohort, AEs were more commonly reported with ADA (50% of total prescriptions), CERT and IXK (both 33.3% of total prescriptions) (Table 10).

An extensive list of AEs and SAEs, categorized according to the MedDRA, is presented in Table 9 and Table 10.

## 4. Discussion

Over the last decade, the therapeutic landscape of immune-mediated rheumatic diseases has expanded substantially with the introduction of b/tsDMARD directed against different inflammatory pathways, providing increasingly personalized therapeutic strategies for patients [9,23].

This study analyzed the evolution of prescribing patterns of b/tsDMARDs in a real-world cohort of patients affected by RA, PsA, SpA, and JIA, followed at the Rheumatology Outpatient Clinic of Tropea during two distinct time periods (2018–2019 and 2023–2024).

The results show that the treated population remained largely comparable in terms of the distribution of the main rheumatic diseases, with a predominance of PsA and RA in both cohorts. Similarly, the sex distribution shows a female predominance, consistent with the epidemiology of chronic inflammatory arthritis and, in particular, RA which is known to be more frequent in females than in males [24].

A particularly interesting finding emerging from the analysis is the increase in the proportion of b/tsDMARD-naïve patients in the 2023–2024 period compared with 2018–2019 (66.4% vs. 52%). This result may reflect a growing tendency toward earlier use of advanced therapies, supported by updates in international recommendations from European Alliance of Associations for Rheumatology (EULAR) and Group for Research and Assessment of Psoriasis and Psoriatic Arthritis (GRAPPA), which in recent years have increasingly advocated a more timely therapeutic approach in patients with persistent disease activity despite conventional treatment [24,25].

From the perspective of comorbidities, arterial hypertension was the most frequently observed condition in both cohorts, followed by cardiovascular diseases in the 2018–2019 period and fibromyalgia in the 2023–2024 period. This finding is consistent with the literature, which reports an increased cardiovascular risk in patients with chronic inflammatory rheumatic diseases, attributed both to persistent systemic inflammation and to the presence of traditional cardiovascular risk factors [26].

The increased prevalence of fibromyalgia observed in the more recent cohort may instead reflect greater diagnostic awareness of this condition in recent years. Fibromyalgia is now recognized as a condition frequently associated with inflammatory rheumatic diseases and capable of significantly influencing both the perception of disease activity and the therapeutic response [27].

The analysis of prescribing patterns highlights a substantial transformation in the therapeutic armamentarium available over the five-year period considered. During 2018–2019, treatment was predominantly based on TNFα inhibitors, which accounted for the majority of initial prescriptions, with ETN and ADA being the most frequently used biologic agents. This finding reflects the historical role of anti-TNFα agents as the first class of biologic drugs introduced into clinical practice and their subsequent consolidation over more than two decades of therapeutic use [28].

During the 2023–2024 period, although ETN remained the most frequently prescribed agent, a marked diversification of therapeutic options emerged, with the introduction and increasing use of drugs targeting novel immunological pathways, including IL-17 inhibitors (SEC and IXK), IL-23 inhibitors (GUS and RSK), including IL-6 inhibitors, particularly sarilumab (SAR), as well as Janus kinase (JAK) inhibitors (TFC, BRC, and UPA). The trends observed in our cohort are consistent with recent findings from large Italian real-world data networks such as VALORE, which have documented both the increasing use of b/tsDMARD therapies in immune-mediated inflammatory diseases and the favorable comparative safety profile of these treatments in routine clinical practice [29,30]. The relative reduction in TNF inhibitor use does not necessarily indicate reduced efficacy of this therapeutic class, which remains a cornerstone of treatment for many patients. Rather, it likely reflects the availability of alternative targeted therapies with different mechanisms of action and safety profiles, allowing clinicians to increasingly tailor treatment according to disease phenotype, comorbidities, previous treatment response, and individual patient characteristics. Different b/tsDMARD classes are characterized by distinct safety profiles, which may influence treatment selection in clinical practice. Factors such as infection risk, cardiovascular comorbidities, gastrointestinal manifestations, and laboratory abnormalities may contribute to the choice of a specific therapeutic strategy, highlighting the importance of an individualized risk–benefit assessment. Among these options, IL-6 inhibitors and JAK inhibitors represent examples of therapies whose specific pharmacological characteristics may be considered in the context of individual patient profiles.

IL-6 inhibitors, by blocking IL-6-mediated signaling, are particularly effective in controlling systemic inflammation and may represent a valuable option in patients with inadequate response to TNF inhibitors or with specific clinical features. Likewise, JAK inhibitors, which interfere with intracellular JAK-STAT signaling downstream of multiple cytokine receptors, provide rapid symptom control and the advantage of oral administration, further expanding therapeutic options in routine clinical practice. Therefore, this evolution reflects the progressive expansion of pathophysiological knowledge in immune-mediated diseases and the availability of increasingly personalized therapeutic strategies tailored to the clinical and biological characteristics of individual patients. In this context, precision medicine refers primarily to the integration of clinical phenotype, disease manifestations, comorbidities, and patient characteristics into therapeutic decision-making. Although biomarker-based treatment selection represents an important perspective for improving therapeutic personalization, its application is currently variable and may not always be feasible in routine clinical settings.

The statistically significant changes observed in prescribing trends between the two periods therefore appear consistent with updates in international guidelines and the increasing availability of therapeutic options. In particular, the relative reduction in the use of IFX and ADA, together with the concurrent increase in agents belonging to the anti-IL-17, anti-IL-23, and JAK inhibitor classes, suggests a progressive shift toward therapeutic strategies more strongly oriented toward precision medicine [23].

Another noteworthy finding is the increased use of biosimilars. Biosimilars are biological medicinal products that are highly similar to an already approved reference biologic, with no clinically meaningful differences in quality, efficacy, safety, or immunogenicity. By the end of 2023, biosimilars available for the treatment of rheumatoid arthritis included infliximab, etanercept, adalimumab, rituximab, and, more recently, tocilizumab. In our cohort, the increased prescription of adalimumab and etanercept biosimilars during the 2023–2024 period is consistent with the progressive incorporation of these agents into routine clinical practice. Numerous studies have demonstrated comparable clinical efficacy, pharmacokinetics, immunogenicity, and safety between biosimilars and their reference biologics, supporting their widespread adoption in rheumatology [31,32]. Their increasing use is also likely driven by growing physician confidence, the accumulation of long-term real-world evidence, and healthcare policies promoting cost-effective prescribing to improve the sustainability of healthcare systems. In our regional setting, this prescribing pattern is consistent with the Calabria Regional Decree (DCA No. 327/2023), which recommends the preferential prescription of the biological or biosimilar with the lowest treatment cost clinically appropriate, while preserving the physician’s clinical judgment. The analysis of treatment switches and swaps further highlights a change in therapeutic failure management strategies. During the 2018–2019 period, a high number of within-class switches was observed, predominantly among anti-TNF agents. In contrast, the 2023–2024 period shows a greater tendency toward inter-class swaps, with transitions to drugs characterized by different mechanisms of action. This prescribing pattern is fully consistent with current EULAR recommendations, which support switching to a different immunological target in patients who do not adequately respond to a first biologic agent [24].

Regarding safety, the results show a reduction in the overall frequency of AEs from 31.7% in the 2018–2019 period to 15% in the 2023–2024 period. In parallel, no SAEs were reported in either cohort. Although the observational design of the study does not allow causal inferences, this reduction may be attributable to improved patient selection for advanced therapies, greater clinical experience in treatment management, and the availability of drugs with different safety profiles.

The observed AEs were broadly consistent with those already reported in the literature for these therapeutic classes, predominantly including asthenia, injection-site reactions, skin rash, headache, and gastrointestinal disorders. Moreover, the absence of new safety signals is in line with findings from international registries and post-marketing pharmacovigilance studies concerning both biologic agents and JAK inhibitors [33].

It is noteworthy that, despite the reduction in AEs, the rate of therapeutic inefficacy was higher in the more recent period (19.3% vs. 13.4%). This apparent discrepancy may reflect the increasing clinical complexity of treated patients and the adoption of more stringent therapeutic targets, in line with the treat-to-target approach currently recommended in inflammatory rheumatic diseases [34].

### Strengths and Limitations of the Study

Among the strengths of this study are the availability of real-world data collected in routine clinical practice and the comparison between two distinct historical periods, which allows for a concrete assessment of the evolution of therapeutic strategies. However, the study has some limitations, including its single-center design and the relatively limited sample size, which did not allow detailed disease-specific analyses, particularly for JIA and SpA due to the limited number of patients in these subgroups. Future studies involving larger and more homogeneous cohorts will be required to better explore disease-specific differences in treatment patterns and outcomes. Second, a methodological limitation involving the statistical analysis is the partial overlap between the two study periods, with 31% of patients in the 2023–2024 cohort having been previously evaluated during the 2018–2019 timeframe. Since this represents a dynamic real-world cohort with substantial changes over time due to clinical drop-outs, deaths, and patient migration to other regions or provincial centers, a strictly paired statistical approach was not mathematically feasible. Although the chi-square test was applied by defining each treatment initiation as an independent prescription event reflective of that specific historical and regulatory landscape, the partial clustering of data at the patient level should be considered interpreting the statistical significance.

Furthermore, the different sample sizes and demographic characteristics between the two cohorts represent an additional limitation. These differences reflect the real-world evolution of the patient population followed at our center over time, rather than predefined matched cohorts. Therefore, the results should be interpreted as a description of changing therapeutic strategies and prescribing patterns in routine clinical practice rather than as a direct comparison between two equivalent populations.

## 5. Conclusions

Overall, this study highlights the evolution of prescribing strategies for chronic inflammatory rheumatic diseases between 2018 and 2024. Although TNF-α inhibitors remained a cornerstone of treatment, their use was progressively complemented by biologics targeting the IL-17 and IL-23 pathways and by targeted synthetic therapies, reflecting the expanding therapeutic landscape and a growing emphasis on personalized treatment. In parallel, the increasing adoption of biosimilars supports current strategies aimed at improving healthcare sustainability.

The findings also confirm the favorable real-world safety profile of advanced therapies, with a lower overall frequency of AEs in the more recent cohort and no serious AEs observed during either study period. While larger studies with longer follow-up are needed to further confirm these findings, our results support the ongoing transition toward more targeted, effective, and individualized therapeutic approaches in rheumatology.

## Figures and Tables

**Table 1:** Characteristics of the study cohort during the periods 2018–2019.

Overall Patients (*n* = 202)		
**Age, years**		
*Mean (±SD)*	54.02 ± 11.7	
*Range (minimum–maximum)*	(min 31–max 82)	
*Median age (IQR)*	56 (41.7–60.2)	
*Mean age first biologic therapy, years (±SD)*	43.7 ± 15.6	
**Gender** ***n*** **(%)**		
Female	125 (61.8)	
Male	77 (38.1)	
F/M ratio	1.62	
**Naïve** ***n* (%)**		
***Naïve***	105 (52)	
Female	65 (61.9)	
Male	40 (38.1)	
F/M ratio	1.62	
***Smoking* ** ***n* ** **(%)**		
Smoker	64 (31.7)	
Ex-smoker	5 (2.47)	
Non-smoker	133 (65.8)	
***Disease* *n* (%)**	***Pt. Naïve, n* (%)**	***Pt. Total, n* (%)**
*Rheumatoid arthritis*	49 (46.7)	91 (45.0)
*Psoriatric arthritis*	56 (53.3)	104 (51.5)
*Juvenile idiopathic arthritis*	0	0
*Anchylosing spondylitis*	0	7 (3.5)
***Comorbidities* *n* (%)**	***Pt. Naïve, n* (%)**	***Pt. Total, n* (%)**
Hypertensive disease	46 (43.8)	98 (48.5)
Thyroid gland disorders	10 (9.4)	19 (9.4)
Cardiovascular disease	19 (18.1)	36 (17.8)
Diabetes	12 (11.4)	24 (11.9)
Hypercholesterolemia	14 (13.3)	24 (11.9)
Fibromyalgia	16 (15.2)	30 (14.8)
Osteoporosis	5 (4.8)	8 (4)
GI manifestations	10 (9.5)	18 (8.9)

Abbreviations: F/M, Female/Male; GI, Gastrointestinal; IQR, Interquartile range; *n*, number; SD, Standard Deviation.

**Table 2:** Characteristics of the study cohort during the period 2023–2024.

Overall Patients (*n* = 140)		
**Age, years**		
*Mean (±SD)*	51 ± 8.92	
*Range (minimum–maximum)*	(min 22–max 80)	
*Median age (IQR)*	51 (42–59)	
*Mean age first biologic therapy, years (±SD)*	48.1 ± 9.5	
**Gender *n* (%)**		
Female	90 (64.3)	
Male	50 (35.7)	
F/M ratio	1.8	
**Naïve *n* (%)**		
Naïve	93 (66.43)	
Female	58 (41.4)	
Male	35 (25)	
F/M ratio	1.65	
***Smokingn* (%)**		
Smoker	26 (18.6)	
Ex-smoker	0 (0)	
Non-smoker	114 (81.4)	
***Disease* *n* (%)**	***Pt. Naïve, n* (%)**	***Pt. Total, n* (%)**
*Rheumatoid arthritis*	42 (30)	64 (45.7)
*Psoriatric arthritis*	51(54.8)	73 (52.1)
*Juvenile idiopathic arthritis*	0	1 (0.7)
*Anchylosing spondylitis*	0	2 (1.4)
***Comorbidities* *n* (%)**	***Pt. Naïve, n* (%)**	***Pt. Total, n* (%)**
Hypertensive disease	48 (34.3)	71 (50.7)
Thyroid gland disorders	9 (6.4)	13 (9.3)
Cardiovascular disease	17 (12.1)	25 (17.8)
Diabetes	9 (6.4)	14 (10)
Fibromyalgia	16 (11.4)	28 (20)
Osteoporosis	3 (2.1)	3 (2.1)
GI manifestations	8 (5.7)	16 (11.4)

Abbreviations: F/M, Female/Male; GI, Gastrointestinal; IQR, Interquartile range; *n*, number; SD, Standard Deviation.

**Table 3:** Treatment Patterns, biosimilar use, treatment switching, concurrent treatment and AEs in patients receiving b/tsDMARDs during the 2018–2019 study period.

Overall Patients (*n* = 202)	
**Biologic drugs prescribed, *n* (%)**	
*ADA*	*34 (16.8%)*
*ETN*	*38 (18.81%)*
*GOL*	*21 (10.4%)*
*IFX*	*23 (11.4%)*
*ABT*	*27 (13.4%)*
*CERT*	*11 (5.4%)*
*SEC*	*11 (5.4%)*
*TCL*	*24 (11.8%)*
*UST*	*6 (3%)*
**Biosimilar, *n* (%)**	
*ADA*	*3 (1.5%)*
*IFX*	*4 (2%)*
*ETN*	*3 (1.5%)*
**Concurrent non-Biologic drugs prescribed, *n* (%)**	
CCs	*29 (14.3%)*
CYA	*6 (3%)*
HCQ	*10 (4.9%)*
MTX	*69 (34.16%)*
SSZ	*4 (2%)*
**Switched, *n* (%)**	
Switch	*54 (26.7%)*
Swap	*13 (6.4%)*
**Adverse events *n* (%)**	
AEs	*64 (31.68%)*
SAEs	*0 (0)*
Inefficacy	*27 (13.4%)*

Abbreviations: ETN, Etanercept; ADA, Adalimumab; ABT, Abatacept; TCL, Tocilizumab, IFX, Infliximab; GOL, Golimumab; CERT, Certolizumab; SEC, Secukinumab; UST, Ustekinumab; CCs, corticosteroids; CYA, cyclosporine A; HCQ, Hydroxychloroquine; MTX, Methotrexate; SSZ, Sulfasalazine; AEs, Adverse Events, SAEs, Serious Adverse Events; *n*, number.

**Table 4:** Treatment patterns, biosimilar use, treatment switching, and AEs in patients receiving b/tsDMARDs during the 2023–2024 study period.

Overall patients (*n* = 140)	
**Biologic drugs prescribed, *n* (%)**	
*ABT*	*13 (9.3%)*
*ADA*	*10 (7.1%)*
*ETN*	*30 (21.4%)*
*GOL*	*12 (8.6%)*
*BRC*	*6 (4.3%)*
*CERT*	*6 (4.3%)*
*GUS*	*5 (3.57%)*
*IFX*	*1 (0.7%)*
*IXK*	*6 (4.3%)*
*RSK*	*2 (1.4%)*
*RTX*	*2 (1.4%)*
*SEC*	*10 (7.1%)*
*SAR*	*1 (0.7%)*
*TCL*	*4 (2.8%)*
*UPC*	*6 (4.3%)*
**Biosimilar, *n* (%)**	
*ADA*	*6 (66.6%)*
*IFX*	*1 (0.7%)*
*ETN*	*6 (17.1%)*
**Concurrent non-Biologic drugs prescribed *n* (%)**	
CCs	50 *(35.7%)*
CYA	4 *(2.8%)*
HCQ	5 *(3.6%)*
MTX	59 *(42.1%)*
SSZ	2 (1.4%)
**Switched, *n* (%)**	
Switch	*8*
Swap	*18*
**Adverse events, *n* (%)**	
AEs	*21 (15%)*
SAEs	*0*
Inefficacy	*27 (19.3%)*

Abbreviations: ETN, Etanercept; ADA, Adalimumab; ABT, Abatacept; TCL, Tocilizumab, IFX, Infliximab; GOL, Golimumab; CERT, Certolizumab; SEC, Secukinumab; BRC, Baricitinib; IXK, Ixekizumab; UPC, Upadacitinib, GUS, Guselkumab GUS; RSK, Risankizumab; RTX, Rituximab; SAR, Sarilumab; CCs, corticosteroids; CYA, cyclosporine A; HCQ, Hydroxychloroquine; MTX, Methotrexate; SSZ, Sulfasalazine; AEs, Adverse Events, SAEs, Serious Adverse Events; *n*, number.

**Table 5:** Switch patterns from initial b/tsDMARD therapy during the 2018–2019 study period.

	Switch to								
Switch from	ADA	ETN	GOL	IFX	ABT	CERT	SEC	TCL	UST
**ADA**		2	2	1	1	3	1	1	1
**ETN**	3		2	1	2	1	1	1	1
**GOL**	-	2		1	-	-	1	-	-
**IFX**	1	5	3		1	-	1	1	1
**ABT**	-	-	-	1		1	-	2	-
**CERT**	-	-	-	-	1		1	1	-
**SEC**	-	-	-	-	-	-		-	-
**TCL**	-	1	1	-	1	-	-		-
**UST**	1	-	-	-	-	-	2	-	

Abbreviations: ADA, Adalimumab; ETN, Etanercept; GOL, Golimumab; IFX, Infliximab; ABT, Abatacept; CERT, Certolizumab; SEC, Secukinumab; TCL, Tocilizumab; UST, Ustekinumab.

**Table 6:** Swap patterns from initial b/tsDMARD therapy during the 2018–2019 study period.

	Swap to								
Swap from	ADA	ETN	GOL	IFX	ABT	CERT	SEC	TCL	UST
**ADA**		2	1	-	2	-	-	-	1
**ETN**	1		1	-	-	-	-	-	-
**GOL**	1	-		-	-	-	-	1	1
**IFX**	1	-	-		-	-	-	-	-
**ABT**	1	-	-	-		-	-	-	-
**CERT**	-	-	-	-	-		-	-	-
**SEC**	-	-	-	-	-	-		-	-
**TCL**	-	-	-	-	-	-	-		-
**UST**	-	-	-	-	-	-	-	-	

Abbreviations: ADA, Adalimumab; ETN, Etanercept; GOL, Golimumab; IFX, Infliximab; ABT, Abatacept; CERT, Certolizumab; SEC, Secukinumab; TCL, Tocilizumab; UST, Ustekinumab.

**Table 7:** Switch patterns from initial b/tsDMARD therapy during the 2023–2024 study period.

	Switch to															
Switch from	ABT	ADA	BRC	CERT	ETN	GOL	GUS	IFX	IXK	RSK	RTX	SAR	SEC	TCL	TFC	UPC
**ABT**		-	-	-	-	-	-	-	-	-	-	-	-	-	-	-
**ADA**	-		-	1	-	-	-	-	-	-	-	-	-	-	-	-
**BRC**	-	-		-	-	-	-	-	-	-	-	-	-	-	-	-
**CERT**	-	-	-		1	-	-	-	-	-	-	-	-	-	-	-
**ETN**	-	3	-	2		1	-	-	-	-	-	-	-	-	-	-
**GOL**	-	-	-	-	-		-	-	-	-	-	-	-	-	-	-
**GUS**	-	-	-	-	-	-		-	-	-	-	-	-	-	-	-
**IFX**	-	-	-	-	1	-	-		-	-	-	-	-	-	-	-
**IXK**	-	-	-	-	-	-	-	-		-	-	-	-	-	-	-
**RSK**	-	-	-	-	-	-	-	-	-		-	-	-	-	-	-
**RTX**	-	-	-	-	-	-	-	-	-	-		-	-	-	-	-
**SAR**	-	-	-	-	-	-	-	-	-	-	-		-	-	-	-
**SEC**	-	-	-	-	-	-	-	-	-	-	-	-		-	-	-
**TCL**	-	-	-	-	-	-	-	-	-	-	-	-	-		-	-
**TFC**	-	-	-	-	-	-	-	-	-	-	-	-	-	-		-
**UPC**	-	-	-	-	-	-	-	-	-	-	-	-	-	-	-	

Abbreviations: ABT, Abatacept; ADA, Adalimumab; BRC, Baricitinib; CERT, Certolizumab; ETN, Etanercept; GOL, Golimumab; GUS, Guselkumab; IFX, Infliximab; IXK, Ixekizumab; RSK, Risankizumab; RTX, Rituximab; SAR, Sarilumab; SEC, Secukinumab; TCL, Tocilizumab; TFC, Tofacitinib; UPC, Upadacitinib.

**Table 8:** Swap patterns from initial b/tsDMARD therapy during the 2023–2024 study period.

	Swap to															
Swap from	ABT	ADA	BRC	CERT	ETN	GOL	GUS	IFX	IXK	RSK	RTX	SAR	SEC	TCL	TFC	UPC
**ABT**		1	-	-	-	-	-	-	-	-	-	-	-	-	-	-
**ADA**	-		1	-	-	-	1	-	-	-	-	-	-	-	-	1
**BRC**	1	1		-	-	-	-	-	-	-	-	-	-	-	-	-
**CERT**	-	-	-		-	-	-	-	-	-	-	-	-	-	-	-
**ETN**	-	-	-	-		-	-	-	-	-	-	-	-	-	-	-
**GOL**	-	-	-	-	-		-	-	-	-	-	-	-	-	-	-
**GUS**	-	-	-	-	-	2		-	1	-	-	-	-	-	-	-
**IFX**	-	-	-	-	-	-	-		-	-	-	-	-	-	-	-
**IXK**	-	-	-	-	-	-	-	-		2	-	-	-	-	-	-
**RSK**	-	-	-	-	-	-	-	-	1		-	-	-	-	-	-
**RTX**	-	-	-	-	-	-	-	-	-	-		-	-	-	-	-
**SAR**	-	-	-	-	-	-	-	-	-	-	-		-	-	-	-
**SEC**	-	-	-	-	-	-	-	-	-	-	-	-		-	-	1
**TCL**	-	-	-	-	-	-	-	-	-	-	-	-	-		-	-
**TFC**	-	-	-	-	-	2	1	-	-	-	-	-	1	-		-
**UPC**	-	-	-	-	-	-	-	-	-	1	-	-	-	-	-	

Abbreviations: ABT, Abatacept; ADA, Adalimumab; BRC, Baricitinib; CERT, Certolizumab; ETN, Etanercept; GOL, Golimumab; GUS, Guselkumab; IFX, Infliximab; IXK, Ixekizumab; RSK, Risankizumab; RTX, Rituximab; SAR, Sarilumab; SEC, Secukinumab; TCL, Tocilizumab; TFC, Tofacitinib; UPC, Upadacitinib.

**Table 9:** MedDRA classification of AEs associated with biologic therapies in 2018–2019.

MedDRA-Compliant Description AEs	ADA	ETN	GOL	IFX	ABT	CERT	TCL	Total
**SOC—General disorders and administration site conditions**	**2**	**4**	**1**	**2**		**1**	**1**	**11**
PT2—Administration site reaction	1	2	1					4
PT3—Hyperthermia		1		1				2
PT4—Hyperhidrosis		1						1
PT5—Asthenia	1			1		1	1	4
**SOC—Skin and subcuta-neous tissue disorders**	**1**	**1**		**2**				**4**
PT1—Rash	1	1		2				4
**SOC—Ear and labyrinth disorders**		**1**		**1**				**2**
PT1—Hypoacusis		1						1
PT2—Vertigo				1				1
**SOC—Nervous system disorders**	**1**	**1**	**1**					**3**
PT2—Headache		1						1
PT3—Demyelination			1					1
PT3—Confusional state	1							1
**SOC—Infections and infestations**	**1**		**1**					**2**
PT—Pneumonia viral	1							1
PT2—Herpes simplex			1					1
**SOC—Respiratory, thoracic and mediastinal disorders**	**1**			**1**			**1**	**3**
PT2—Dyspnoea	1						1	2
PT3—Throat tightness				1				1
**SOC—Investigations**						**1**	**1**	**2**
PT1—Hepatitis C antibody positiveal							1	1
PT2—Transaminases increased						1		1
**SOC—Blood and lymphatic system disorders**					**1**			**1**
PT—Thrombocytopenia					1			1
**SOC—Gastrointestinal disorders**	**1**			**2**				**3**
PT—Nausea	1			1				2
PT2—Vomiting				1				1
**SOC—Eye disorders**	**1**							**1**
PT—Visual impairment	1							1
**Total AEs**	**16**	**14**	**6**	**16**	**2**	**4**	**6**	**64**

Adverse event classes are shown in bold to distinguish them from their respective subclasses. Only drugs associated with at least one adverse event are reported in the table. Abbreviations: ADA, Adalimumab; ETN, Etanercept; GOL, Golimumab; IFX, Infliximab; ABT, Abatacept; CERT, Certolizumab; TCL, Tocilizumab.

**Table 10:** MedDRA classification of AEs associated with biologic therapies in 2023–2024.

MedDRA-Compliant Description AEs	ABT	ADA	CERT	ETN	IXK	TFC	UPC	Total
**SOC—General disorders and administration site conditions**		**3**	**1**	**1**	**1**	**1**	**1**	**8**
PT1—Asthenia			1		1	1		**3**
PT4—Administration site reactions		2		1				**3**
PT6—Pyrexia		1					1	**2**
**SOC—Skin and subcutaneous tissue disorders**		**1**	**1**	**1**				**3**
PT2—Pruritus		1	1	1				**3**
**SOC—Ear and labyrinth disorders**	**2**							**2**
PT—Vertigo	2							2
**SOC—Nervous system disorders**	**1**			**1**				**2**
PT—Headache	1			1				2
**SOC—Respiratory, thoracic and mediastinal disorders**					**1**	**1**		**2**
PT2—Nasopharyngitis					1			1
PT3—Bronchospasm						1		1
**SOC—Gastrointestinal disorders**						**2**		**2**
PT2—Nausea						1		1
PT3—Diarrhea						1		1
**SOC—Musculoskeletal and connective tissue disorders**		**1**		**1**				**2**
PT—Limb discomfort				1				1
PT1—Myalgia		1						1
**Total AEs**	**3**	**5**	**2**	**4**	**2**	**4**	**1**	**21**

Adverse event classes are shown in bold to distinguish them from their respective subclasses. Only drugs associated with at least one adverse event are reported in the table. Abbreviations: ABT, Abatacept; ADA, Adalimumab; CERT, Certolizumab; ETN, Etanercept; IXK, Ixekizumab; TFC, Tofacitinib; UPC, Upadacitinib.

## Data Availability

The data supporting the findings of this study are contained within the article. Individual patient data are not publicly available due to privacy and confidentiality restrictions.

## Abbreviations

The following abbreviations are used in this manuscript:

ABT	Abatacept
ADA	Adalimumab
AEs	Adverse events
AS	Anchylosing spondylitis
ASP	Provincial Health Authority
BRC	Baricitinib
b/tsDMARDs	Biologic and targeted synthetic disease-modifying antirheumatic drugs
CERT	Certolizumab
CCS	Corticosteroids
CyA	Cyclosporine A
DMARDs	Disease-modifying antirheumatic drugs
ETN	Etanercept
EULAR	European Alliance of Associations for Rheumatology
GOL	Golimumab
GRAPPA	Group for Research and Assessment of Psoriasis and Psoriatic Arthritis
GUS	Guselkumab
HCQ	Hydroxychloroquine
JAK	Janus kinase
JIA	Juvenile idiopathic arthritis
IFX	Infliximab
IQR	Interquartile range
IL	Interleukin
IL-6	Interleukin-6
IL-17	Interleukin-17
IL-23	Interleukin-23
AIFA	Italian Medicines Agency
IXK	Ixekizumab
MedDRA	Medical Dictionary for Regulatory Activities
MTX	Methotrexate
RNF	National Pharmacovigilance Network
PsA	Psoriatic arthritis
RA	Rheumatoid arthritis
RSK	Risankizumab
RTX	Rituximab
SAR	Sarilumab
SEC	Secukinumab
SAEs	Serious Adverse Events
SSZ	Sulfasalazine
SpA	Spondyloarthritis
TCL	Tocilizumab
TFC	Tofacitinib
UPC	Upadacitinib
UST	Ustekinumab
